# Bacterial Stress Responses: What Doesn't Kill Them Can Make Them Stronger

**DOI:** 10.1371/journal.pbio.0040023

**Published:** 2006-01-17

**Authors:** Kathryn J Boor

## Abstract

Identifying specific mechanisms that contribute to microbial survival under rapidly changing conditions could provide insight into stress response systems across life forms.

An organism's survival from moment to moment depends, at least in part, on its ability to sense and respond to changes in its environment. Mechanisms for responding to environmental changes are universally present in living beings. For example, when mammals perceive a sudden environmental change as threatening, a rush of adrenaline precipitates the well-known “fight or flight” response. Such physiological stress responses in complex organisms require appropriately regulated interactions among numerous organ systems. But how do single-celled organisms respond to potentially lethal threats? The hope is that identifying specific mechanisms that contribute to microbial survival under rapidly changing conditions will provide insight into stress response systems across life forms.

Bacteria—and especially those capable of persisting in diverse environments, such as Escherichia coli—provide particularly valuable models for exploring how single-celled organisms respond to environmental stresses. For example, most bacteria associated with foodborne infections (e.g., some E. coli serotypes, Salmonella enterica serovar Typhimurium, Listeria monocytogenes) can survive under diverse conditions, both inside and outside of the host. To ultimately cause human infection, a foodborne pathogen must first survive transit in food or water, a significant achievement since the majority of commercial products destined for consumption in the United States are treated with strategies specifically designed to control or eliminate microbial contaminants. Following ingestion, the bacterium must survive exposure to conditions that have evolved to provide the host with some protection against pathogenic microbes. Human bodily defenses include gastric acid (ranging from [pH 2.5–4.5], largely depending on feeding status), bile salts, and organic acids within the gastrointestinal tract. To survive these extreme and rapidly changing conditions, bacteria must sense the changes and then respond with appropriate alterations in gene expression and protein activity. Therefore, one important scientific challenge is to identify mechanisms that control the switch or switches that allow free-living bacteria to adjust to and invade a host organism.

## The Role of Sigma Factors in Transcription

In bacteria, alterations in gene expression are often controlled at the transcriptional level through changes in associations between the catalytic core of RNA polymerase and the different sigma factors present in a bacterial cell [1]. RNA polymerase is the enzyme responsible for recognizing appropriate genes under specific environmental conditions, and for creating the mRNA transcripts that can be translated into new proteins. Sigma factors are dissociable subunits of prokaryotic RNA polymerase. When a sigma factor associates with a core RNA polymerase to form RNA polymerase holoenzyme, it directs the holoenzyme to recognize conserved DNA motifs called promoter sites (or regions) that precede gene sequences. Sigma factors also contribute to DNA strand separation, which is a critical step in transcription initiation. The sigma subunit dissociates from the RNA polymerase core enzyme shortly after transcription begins, thus becoming available for reassociation. Associations between different alternative sigma factors and core RNA polymerase essentially reprogram the ability of the RNA polymerase holoenzyme to recognize different promoter sequences and express entirely new sets of target genes. As the set of genes controlled by a single sigma factor (also known as the regulon) can number in the hundreds, sigma factors provide effective mechanisms for simultaneously regulating large numbers of prokaryotic genes.

How do single-celled organisms respond to potentially lethal threats?

Sigma factors are classified into two structurally unrelated families: σ^54^ and σ^70^ families. Subunits comprising the σ^54^ family are often commonly referred to as σ^N^. σ^N^ has been identified in multiple diverse species, including Legionella pneumophila, Pseudomonas spp., Enterococcus faecalis, Campylobacter jejuni, and L. monocytogenes. In addition to regulating nitrogen metabolism in a number of organisms, σ^N^-dependent genes also contribute to a diverse array of metabolic processes [2,3]. The σ^70^ family, which is larger and more diverse than the σ^54^ family, is divided into four groups based on conservation of their primary sequences and structures [4,5]. The Group I sigma proteins are the primary sigma factors (e.g., Bacillus subtilis σ^A^) and are also referred to as “housekeeping” sigma factors, as they direct transcription of genes important for bacterial growth and metabolism. Sigma factors in the remaining groups are also referred to as alternative sigma factors [6] and often regulate specific physiological processes, e.g., sporulation. σ^70^ family members that contribute to bacterial stress responses (e.g., σ^S^, σ^B^, and some extracytoplasmic function sigma factors) are of particular interest as mounting evidence suggests that in bacterial pathogens, these regulatory proteins serve as links between bacterial abilities to respond to changes imposed by the host environment and, subsequently, to cause disease (e.g., [7,8]).

Bacteria are classified as Gram-negative or Gram-positive based on microscopically observed staining properties associated with different cell membrane structures. σ^S^ (RpoS) and σ^B^ (SigB) have been identified as general stress responsive alternative sigma factors in Gram-negative and in Gram-positive bacteria, respectively. σ^S^ was identified in both E. coli and in *S.* Typhimurium as a Group II sigma factor that activates expression of numerous genes required to maintain cell viability as the cell leaves exponential growth conditions and moves into stationary phase [9,10]. In addition to helping E. coli and *S.* Typhimurium respond to different environmental stress conditions, such as those associated with entry into stationary phase, σ^S^ also contributes to expression of virulence-associated genes [10]. Since its initial discovery, the presence of σ^S^ and its role in stress response has been confirmed in multiple, diverse Gram-negative bacterial pathogens, including Pseudomonas aeruginosa, L. pneumophila, Borrelia burgdorferi, Yersinia enterocolitica, and Shigella flexneri.

σ^B^, a Group III sigma factor encoded by *sigB*, was initially identified and characterized in B. subtilis [11,12], but has also been identified in L. monocytogenes, Staphylococcus aureus, B. anthracis, and B. licheniformis. The B. subtilis σ^B^-dependent general stress regulon is large: over 200 genes are expressed following bacterial exposure to heat, acid, ethanol, salt stress, entry into stationary phase, or starvation for glucose, oxygen, or phosphate [13,14]. While disruption of *sigB* in B. subtilis has no apparent effect on the organism's ability to sporulate or to grow under many conditions, *sigB* mutants are sensitive to oxidative stress [15], and exhibit impaired growth in ethanol and reduced survival at extreme pH [16]. L. monocytogenes sigB mutants are more sensitive than wild type to acid and oxidative stress, as well as to nutrient depletion [17]. In L. monocytogenes, σ^B^ contributes to expression of internalin A and internalin B, two bacterial surface-associated proteins important for host-cell invasion [7,8].

The extracytoplasmic function (ECF) Group IV sigma factors are conserved across both Gram-positive and Gram-negative species [18], and comprise a large, phylogenetically distinct subfamily within the σ^70^ family. σ^E^, an ECF sigma factor that was initially recognized as a heat-shock sigma factor in E. coli [19], responds to accumulation of specific unfolded proteins in the periplasm [20]. Members of the ECF subfamily are distinct from the rest of the σ^70^ family in that they regulate a wide range of functions involved in sensing and reacting to conditions in the membrane, periplasm, or extracellular environment [21]. Sensing of the extracellular environment is achieved via a signal transduction mechanism in which the ECF sigma factor is bound to a cognate inner membrane–bound anti-sigma factor.

## The Proteins Regulated by Sigma Factors

To fully understand the biological contributions of regulatory proteins such as sigma factors, it is critically important to identify genes regulated by these proteins. To date, investigators have used combinations of global (e.g., computer-based sequence similarity searches for conserved promoter sequences, two-dimensional protein gel electrophoresis, microarray analyses) and more focused strategies (e.g., in vitro transcription methods, reporter fusion transposon mutagenesis) to identify sigma factor regulons (e.g., [13,14,22,23]). In the study by Carol Gross and her colleagues [24], published in this issue of *PLoS Biology*, σ^E^-regulated transcription units were identified in E. coli K-12 through multiple strategies, including microarray profiling and rapid amplification of cDNA ends, as well as by using a sophisticated computer-based DNA motif search strategy that was designed using sigma E promoter consensus sequence data garnered by the team from E. coli [24]). The authors then used their computer-based search strategy to identify potential sigma E motifs upstream of genes in E. coli and in eight additional Gram-negative genera. Broadly speaking, one exciting outcome of this work is the development of an effective set of bioinformatic tools that will be useful in mining DNA sequence databases for the presence of conserved sequences by allowing more rapid and accurate prediction of genes that are coordinately regulated.

The results reported by Rhodius et al. [24] unambiguously confirm the role of σ^E^ in maintenance of the integrity of the bacterial cell's outer membrane, but they also highlight a critical role for σ^E^ in regulating expression of virulence-associated genes among the pathogenic bacteria included in their study (e.g., E. coli O157:H7, *S.* Typhimurium, S. flexneri). These data suggest the possibility that σ^E^, which is important for bacterial responses at the cell surface, may represent an important switch mechanism that facilitates bacterial transition from a free-living organism to a host-invading pathogen. Studies of this nature provide powerful new insight into the field of microbial physiology by enabling rapid identification of genes that may appear to be unrelated in function, but that must be coordinately regulated to enable an organism to survive and respond appropriately under rapidly changing environmental conditions, such as those encountered by a bacterial pathogen during the infection process. These coordinately regulated genes ultimately may prove to be appropriate targets for development of novel antimicrobial strategies, thus providing tangible realization of the promise and power of the application of genomics tools for improving human health.

## Abbreviation

ECF: extracytoplasmic function
